# Effect of videotape for home instruction on the quality of life of tracheostomy patients: a randomized clinical trial

**Published:** 2015

**Authors:** N Mohammadi, M Farahani, S Vatandost

**Affiliations:** * Faculty of Nursing and Midwifery, Iran University of Medical Sciences, Tehran, Iran; ** Faculty of Nursing and Midwifery, Kurdistan University of Medical Sciences, Sanandaj, Iran

**Keywords:** videotape, quality of life, tracheostomy patients

## Abstract

**Background:** Nowadays, some of the patients, such as the patients with tracheostomy are taken care of at home. The quality of life of these cases may decrease due to the appearance of changes, limitations caused by tracheostomy and improper care.

**Objectives:** This research was performed in order to discover the effect of videotape for home instruction on the quality of life of tracheostomy cases.

**Patients and Procedure:** A quasi-empirical research was carried out on 80 cases by a tracheotomy in Amir Elm Hospital and the Imam Khomeini Medical Center in Tehran. Patients were randomized into two teams of invasion and check through the randomized block design method. At the time of discharge and after a routine training from the clinical staff, the two groups completed a questionnaire regarding the demographic information and the sf-36 quality of life. Then, the intervention team was given a CD containing the routine training for patient care to watch at home. After two months, the participants completed the sf-36 questionnaires again, and the data were entered into SPSS version 20. The information was investigated by using the statistical tests.

**Findings:** The statistical investigation demonstrated that the comparison between the two groups after the intervention in the overall quality of life (p = 0.003) and all the concepts included: the physical role (p = 0.02), the mental role (p = 0.01), energy and fatigue (p = 0.03), motion health (p = 0.005), social functioning (p = 0.06), disorder (p = 0. 001), common health percentage (p = 0.002) and physical functioning (p = 0.001) in the arbitration team, being higher than in the check one.

**Conclusion:** Using the videotape education additionally to routine care has a significant effect on the improvement of the quality of life in these patients. Therefore, the use of this method is recommended as a complementary program for tracheostomy patients.

## Introduction

The increase in hospitalization costs and being far away from family and nosocomial infections has made looking after patients at home more welcomed [**1**]. Tracheostomy represents the creation of a gap or valve on the trachea to provide an airway in patients with upper respiratory system obstruction caused by tumors of the larynx, thyroid, and esophagus. Larynx preservation is common in patients who require long-term intubation [**2**,**3**]. In the lack of proper care, complications of tracheostomy include infection, tube duct obstruction due to lack of proper cleaning and accidental extubation of the tracheostomy tube [**4**]. What should also be considered are the new circumstances in their lives, including an unpleasant change in their appearance and the limitations due to a tracheostomy. This group of patients may suffer from stress, low self-esteem, negative change in their appearance [**5**], difficulty in communicating effectively with others and isolation and, as a result, these can have an adverse effect on their quality of life [**6**].

There is no comprehensive agreement on the description of the quality of life [**7**]. However, according to the definition provided by the World Health Organization, quality of life represents people’s understanding of their situation in life regarding culture, the value system of the area they live in, goals, expectations, standards, and their priority. Therefore, the topic is entirely individual and subjective, not observable by other people and according to the personal understanding of different aspects of his or her life [**8**]. The quality of life contains various dimensions including physical, mental, and social functioning, understanding and perception of each person’ wellbeing and health, disability and lifetime [**9**]. Researchers believe that the investigation of the quality of life and the efforts to improve it has a main effect on the health of the patient’s personal and social life [**10**]. Additionally, the quality of life condition has always been considered as being in accordance with the findings in clinical studies, treatment, and health care [**11**]. Typically, hospitalized patients admitted with any diagnosis, receive routine instructions during hospitalization and discharge, which is common in healthcare centers [**12**]. The main groups involved in the education are nurses [**13**]. In most of the cases, this training, which is mostly done orally and face to face, makes the process of instruction problematic due to having a boring, energy-intensive, and time-consuming method, and it needs many tactful, experienced instructors [**14**]. Results of a study conducted by Deccache et al. in 2012 indicated that only 20% of the hospitalized patients are satisfied with the provided information on the illness and its treatment, while, 60% of them expected more and better information and 20% were completely unsatisfied with the training program [**15**]. These problems may increase the disease’s complications, readmissions and care expenses [**16**]. Furthermore, patients can be active in the process of the illness by gaining self-care skills through a proper training on their safety, performance ability [**17**]. There are varieties of methods to provide education, and it is important that trainers choose the best way to instruct [**18**]. One of the educational resources is to use videotape tools, for which the practical impact, as a good way to generate interest and increase learning, has been proven [**19**]. The benefits of video training are the ability to create storage for a lot of information, creating continuity in the data, the absence of anxiety during training and the capability of adding new information to old ones [**20**]. The other benefit of using video training is using color, motion, and different scenes, which all come with the audio training and represent an inclusive education [**21**]. Furthermore, this method is inexpensive and affordable [**22**].

Reviewing the previous studies, they showed that the impact of audio and video training in promoting quality of life is positive, such as a study by Baraz-Pardenjani showed that the utilization of instructional videos raises the quality of life in hemodialysis cases [**23**]. The survey results of Sheikh et al. also revealed that audio and video training is effective in improving the quality of life in cases with type II diabetes [**24**]. The study of Vocht et al. indicated that training with the help of video causes an improvement in the quality of life of elderly patients suffering from psychological issues [**25**]. Because tracheostomy patients require long-term attention at home, self-care training can be beneficial in reducing the incidence of tracheostomy complications thus increasing the quality of life. Therefore, the conducted research intended to survey the effect the education videotape has on the quality of life of the cases with tracheostomy.

**Patients and Procedures**

This study is a quasi-experimental one involving two groups, which is the result of a master program thesis approved at the University of Tehran Medical Sciences. This study was registered in the clinical trial database www.Irct.ir.

**Participants**

The participants were patients with a tracheostomy in the ENT ward of Amir Alam Hospital and the Imam Khomeini Institute of Cancer in Tehran. 

**Inclusion Criteria**

Inclusion criteria included: patients over 20 years old, self-care ability, cooperative ability, not having a history of mental illness, full awareness of the time, place and the person at the time of training, completing the questionnaires, access to facilities for watching training videos, lack of experience or training in health centers and the ability to communicate in Persian language.

**Exclusion Criteria**

Unwillingness to continue cooperation for any reason, deterioration of the person’s condition and inability to complete the questionnaire, lack of possibility to coordinate an appointment to fill out the questionnaire after the intervention.

**Tools**

The instrument used to gather and record the information in this research included a survey that had three parts and included information about the study and intervention explanation, demographic information and SF-36 quality of life questionnaire [**26**]. The validity of the content of population data was conducted through a literature review. Afterwards, it was studied by ten professors, and their feedbacks were applied. The SF-36 quality of life survey included 36 questions that investigated eight aspects of life (role limitations because of physical health issues, role restriction because of mental health issues, vitality (energy, fatigue), general emotional health, social functioning, bodily disorder, common health realization, and physical functions). Scores on each scale varied from 0 to 100. Zero proposed the worst and 100 proposed the optimal condition in the report. The translation of SF-36 quality of life survey to Persian language and also its reliability and validity evaluation were performed by Montazeri and colleagues in 2005 [**27**].

**Sampling and intervention**

After the confirmation via the ethics group of Tehran University of Medical Sciences, subjects were chosen though utility sampling, and after obtaining a written informed consent to take part in the research, participants were set into two groups of 5 people based on random block method. In this study, assuming P = 0.5 and the confidence interval of 95%, the total number of samples was 80, and considering the loss, there were 45 people calculated for each group. In the intervention group, one patient was excluded from the study due to the unwillingness to continue participating in the study, and four patients due to defects in their questionnaire. In the control group, five patients avoided completing the survey after two months and were therefore excluded from the comprehensive study. In addition, at the end of the survey, the total number of the participants who completed the questionnaire for each group was 40 people. During discharge, after the regular training of the medical staff, both groups completed the questionnaires mentioned above. In the intervention group, in addition to the regular training received from the medical staff, one educational CD with audio and video features created by the researcher was given to the patients for use at home. The content of the CD contained an introduction to tracheostomy care, showing the daily care the tracheostomy patients need including bathing, shaving, suction, replacing the bandage around tracheostomy, cleaning the tracheostomy tube, the tracheostomy site infection symptoms, how to communicate with others and the way they should present in public. The scientific confirmation of the content of the film was given by ten professors from Tehran University of Medical Sciences. The intervention group could watch the movie with no restrictions regarding the number of times at home. To ensure that it was watched by the patients, two telephone numbers reminded them of this issue. In addition, all the patients mentioned that they watched the video. What should also be mentioned is that there were no restrictions for other members of the family regarding the watching of the movie. After two months, the patients were coordinated by the two groups to have another meeting to complete the questionnaire.

**Statistical Calculations**

The data were entered into SPSS version 20. Kolmogorov-Smirnov test showed a normal distribution. Thus, an independent t-test was employed to compare the two teams. Paired T-test was employed to make an evaluation before and after a mean of a cluster. The significance level was of less than 05%.

## Results

Most participants were in the age group of 60 years and more. 62.5% of the participants in the check team and 52.5% in the invasion team were men. Most participants in the check group, 52.5% were living with their wife and children, and the invasion team, 62.5% were living with the others who were 15% in the check team and 7.5% in the invasion.

The most common job between the two groups was self-employment, 37.5% in the check team, and 40% in the invasion team. The least common job was unemployment, which was 5% in both groups. The highest level of education in the check team was under diploma, 30%, and, in the invasion team, it was elementary school and a diploma, which included 30% for each one, whereas the lowest education level in the check team was primary education, 20%, and academic education, 15%, in the invasion team.

The income of both groups was at a sufficient degree, being of 47.5% in the check team and of 60% in the invasion team. The most common cause of tracheostomy was a laryngeal tumor, which was 40% in the check team and 35% in the invasion team. The least common cause of tracheostomy was tracheal tumor, which was 5% in the check team and 7.5% in the invasion team. The results of the chi-square and chi-square experiment indicated that there was no clear variation among the demographic characteristics of both groups, and being homogenous regarding population characteristics (**Table 1**).

**Table 1:** Demographic features of check and invasion teams

Variable	control number (percent)	intervention number (percent)	test result (P-value)
Age			
20-30	3 (7/5)	5 (12/ 5)	
30-40	5 (12/ 5)	5 (12/ 5)	
40-50	6 (15)	5 (12/5)	0/593
50-60	7 (17/5)	4 (10)	
60-70	9 (22/5)	11 (27/5)	
Above 70	10 (25)	10 (25)	
Sex			
Male	25 (62/5)	21 (52/5)	
Female	15 (37/5)	19 (47/5)	0/652
People who live with the patient			
Children and wife	21 (52/5)	25 (62/5)	
children	13 (32/5)	12 (30)	0/601
others	6 (15)	3 (7/5)	
Job			
clerk	4 (10)	4 (10)	
Self-employment	15 (37/ 5)	16 (40)	
Retired	9 (22/ 5)	7 (17/ 5)	0/ 661
Housewife	10 (25)	11 (25)	
Workless	2 (5)	2 (5)	
Education			
Primary education	8 (20)	12 (20)	
High school	12 (30)	10 (25)	
Diploma	11 (28/ 5)	12 (30)	0/ 671
College	9 (21/ 5)	6 (15)	
Income			
Enough	10 (25)	10 (25)	
Somewhat enough	19 (47/ 5)	24 (60)	0/ 845
Not enough	11 (27/ 5)	6 (15)	
Cause tracheostomy			
Larynx tumor	16 (40)	14 (35)	
Prolong intubation	8 (20)	4 (10)	
thyroid tumor	7 (17/ 5)	10 (25)	0/ 310
Face and head trauma	4 (10)	4 (10)	
Head and neck surgery	5 (12/ 5)	3 (7/5)	
Esophageal tumor	3 (7/ 5)	2 (5)	

The mean of the overall quality of life and standard deviation before the intervention was 40.46 ± 2.58 in the check team and 40.30 ± 7.25 in the intervention team. The results of independent t-test showed that the two teams were homogeneous before the intervention (P = 0.125). Also, both teams showed no clear distinction in any of the eight concepts of the quality of life (p > 0.05) before the intervention (**Table 2**).

**Table 2:** Comparison between the average rate of the quality of life and the dimensions in the control and intervention group in discharge

Group Dimension of QOL	Control (Mean ± SD)	intervention (Mean ± SD)	Test result (p-value)
Role - Physical	52/ 65 ± 8/ 41	52/ 18 ± 8/ 55	0/ 52
Role - Emotional	36/ 40 ± 5/ 81	36 ± 5/ 55	0/ 31
Vitality (Energy, fatigue)	41/ 71 ± 8/ 41	43/ 90 ± 8/ 43	0/ 23
General Mental health	41/ 71 ± 8/ 41	43/ 90 ± 8/ 43	0/ 23
Social functioning	38/ 75 ± 10/ 88	42/ 18 ± 12/ 33	0/ 33
Bodily Pain	35 ± 9/ 47	39/ 06 ± 10/ 27	0/ 81
General health perception	38/ 62 ± 5/ 88	39/ 75 ± 7/ 15	0/ 78
Physical functioning	38/ 75 ± 10/ 89	42/ 18 ± 12/ 23	0/ 33
Overall QOL	40/ 46 ± 2/ 58	40/ 30 ± 7/ 25	0/ 12

After the intervention, the mean and standard deviation in the control group was 41.93 ± 9.28 and in the intervention group, it was 47.12 ± 9.28. The result of the paired t-test indicated that the two groups were statistically different (P = 0.03). In addition, the mean of the overall quality of life in the inversion team was higher than in the check team. Additionally, in all the dimensions of the quality of life, the two groups were statistically different: role limitations because of feelings issues (p = 0.01), vitality (fatigue, energy) (p = 0.03), general mental health (0.005), social functioning (p = 0.006), bodily disorder (0.001), common health realization (p = 0/ 02) and physical functioning (p = 0/ 01), which were present more in the intervention team than in the check one (**Table 3**).

**Table 3:** Comparison between the average rate of the quality of life and the dimensions in the control and intervention group at 2 months after discharge

Group Dimension of QOL	Control (Mean ± SD)	Intervention (Mean ± SD)	Test result (p-value)
Role - Physical	47/ 7 ± 3/ 92	52/ 71 ± 6/ 50	0/ 02
Role - Emotional	27/ 65 ± 6/ 88	42/ 51 ± 12/ 77	0/ 01
Vitality (Energy, fatigue)	41/ 56 ± 7/ 82	52/ 59 ± 12/ 59	0/ 03
General Mental health	37/ 40 ± 7/ 28	55/ 93 ± 10/ 80	0/ 005
Social functioning	36/ 87 ± 9/ 36	44/ 87 ± 15/ 08	0/ 006
Bodily Pain	36/ 01 ± 9/ 59	47/ 98 ± 15/ 57	0/ 001
General health perception	39/ 62 ± 6/ 73	45/ 57 ± 11/ 45	0/ 002
Physical functioning	36/ 87 ± 9/ 36	45/ 32 ± 15/ 05	0/ 001
Overall QOL	41/ 93 ± 9/ 28	47/ 12 ± 9/ 28	0/ 003

The comparison between the average and the normal deviation of the overall quality of life in the check group in discharge and the two-months later, depicted that variations were not statistically notable (p = 0.09). Additionally, the comparison between the 8 concepts of the quality of life showed the differences in role restriction due to body health issues (p = 0.03), role limitations because of mental issues (p = 0.001), general mental health (p= 0.03), social functioning (p = 0.04) and physical functioning (p = 0.02), being statically significant for the decrease in the quality of life. No clear difference was observed in other concepts, such as vitality (energy/ fatigue) (p = 0.92) and bodily pain (p = 0.16), when a main increase in the general health perception was observed (p = 0.03) (**Table 4**).

**Table 4:** Comparison between the pre and post mean score of Quality of life (QOL) and the dimensions in the control group

Dimension of QOL	Discharge (Mean ± SD)	2 Months later (Mean ± SD)	Test result (p-value)
Role - Physical	52/ 65 ± 8/ 41	47/ 7 ± 3/ 92	0/ 03
Role - Emotional	36/ 40 ± 5/ 81	27/ 65 ± 6/ 88	0/ 001
Vitality (Energy, fatigue)	41/ 71 ± 8/ 41	41/ 56 ± 7/ 82	0/ 92
General Mental health	41/ 71 ± 8/ 41	37/ 40 ± 7/ 28	0/ 03
Social functioning	38/ 75 ± 10/ 88	36/ 87 ± 9/ 36	0/ 04
Bodily Pain	35 ± 9/ 47	36/ 01 ± 9/ 59	0/ 16
General health perception	38/ 62 ± 5/ 88	39/ 62 ± 6/ 73	0/ 03
Physical functioning	38/ 75 ± 10/ 89	36/ 87 ± 9/ 36	0/ 02
Overall QOL	40/ 46 ± 2/ 58	41/ 93 ± 9/ 28	0/ 09

The comparison between the average and normal deviation of the overall quality of life before and after education, in the intervention group, showed that overall quality of life increased from 40.30 ± 7.25 to 47.12 ± 9.28, and the paired t-test revealed that this variation was statistically important (p = 0.001). Additionally, the comparison between the 8 concepts of the quality of life showed the differences in all demotions: role restriction due to mental issues (0.005), role limitations because of physical health concerns, (0.02), general mental health (0.01), physical functioning (0.04) and social functioning (0.02), bodily disorder (0.02), general health perception (0.005), vitality (energy, fatigue) (0.002), being significantly increased (**Table 5**).

**Table 5:** Comparison between the pre and post average rate of the quality of life and the dimensions in the intervention group

Group Dimension of QOL	Discharge (Mean ± SD)	2 Months later (Mean ± SD)	Test result (p-value)
Role - Physical	52/ 18 ± 8/ 55	52/ 71 ± 6/ 50	0/ 02
Role - Emotional	36 ± 5/ 55	42/ 51 ± 12/ 77	0/ 005
Vitality (Energy, fatigue)	43/ 90 ± 8/ 43	52/ 59 ± 12/ 59	0/ 002
General Mental health	43/ 90 ± 8/ 43	55/ 93 ± 10/ 80	0/ 01
Social functioning	42/ 18 ± 12/ 33	44/ 87 ± 15/ 08	0/ 02
Bodily Pain	36/ 09 ± 10/ 27	47/ 98 ± 15/ 57	0/ 02
General health perception	39/ 75 ± 7/ 15	45/ 57 ± 11/ 45	0/ 005
Physical functioning	42/ 18 ± 12/ 23	45/ 32 ± 15/ 05	0/ 04
Overall QOL	40/ 30 ± 7/ 25	47/ 12 ± 9/ 28	0/ 001

## Discussion

In the fast-developing contemporary community, electric health concern regularities are presently the best feasible approach to attain enhanced service productivity and quality [**28**]. This research hypothesized that tracheostomy cases presented to the video education home program guidance would have the most real quality of life criteria associated with tracheostomy cases on regular training. The conclusions of this research pointed out notable developments in the QOL of the cases in the intervention team, which resulted in the approval of the research proposal. The two teams of cases involved in the research were alike regarding all their socio-demographic features. This relationship was necessary to assure that any variations taken after the invasion would not be connected to the variations in these features.

Two months after the discharge from the hospital, the comparison between both groups showed statistically significant differences in the mean score of the overall quality of life and the concepts, that being higher in the intervention group. A research undergone by the State of Iran indicated that the quality of life of patients with a permanent pacemaker after self-care training by video method in the intervention group had significant increases as compared with the ones in the control group. Additionally, the quality of life in emotional, physical, and social concepts was clearly larger in the invasion team [**29**]. A research by Headley et al. in the USA showed that video education might significantly increase the quality of life and concepts in breast cancer cases in the intervention team as compared to the check team [**30**]. A research via Bar Pard Anja Ni in Iran indicated that video education might influence the quality of life and the concepts in hemodialysis patients [**23**]. Mahmoud and Valley revealed that health literacy was sufficient on the quality of life of married women [**31**,**32**]. Also, the study of Salameh et al. in Palestine indicated which electronic program education will have a more positive effect on the quality of life quality in coronary heart problems compared to routine training in the control group [**33**]. The conclusions of this research are compatible with the above-mentioned studies confirming the significant effect of video teaching method on the quality of life.

The comparison between the quality of life and its concepts in discharge and two months later, in the check team, indicated that the overall quality of life increased with 1.47 points in a range of 100, but this increase was not statistically significant. However, in the majority of the concepts, such as role limitations because of bodily fitness issues, role boundaries since mental issues, common psychic health, human functioning, physical functioning, and general health perception, it decreased significantly. In addition, in the vitality (energy/ fatigue) and bodily pain concepts of quality of life, no clear difference was observed. A study by Atlee et al. showed that the quality of life of the cases with permanent pacemakers decreased in mental and social concepts a month after the implantation of a pacemaker and, no significant difference was observed in the physical aspects [**29**]. Furthermore, a study by Hashmi et al. indicated that the quality of life of patients with tracheostomy decreased after the implantation of a tracheostomy in the absence of a proper education [**5**], which was consistent in this research. Therefore, the results of this study and the mentioned studies indicated that chronic patients, such as patients with tracheostomy who only received routine training in health centers for self-care, experienced a decrease in the quality of life; hence, more attention needed to be paid regarding the training of these patients.

The comparison between the quality of life and its concepts before and after the intervention, in the intervention group, showed that the overall quality of life increased with 6.82 points in a range of 100, this increase being statistically significant and also all the concepts of the quality of life increased, thus being statically significant. The study by Baraz- Pardenjani et al. in Iran showed that the use of video education in hemodialysis patients increased the overall quality of life and also increased the bodily functioning, mechanical function, mental role, social functioning and common health ideas [**23**]. A research by Stalker in the United Kingdom indicated that the use of video education in hemophilia patients with different educational background increased the overall quality of life, mental and physical concepts [**34**,**35**]. The finding in this study was in accordance with the studies mentioned earlier. Therefore, we suggest the use of video education in addition to routine training received from the clinical staff members, due to its providing a proper source on the correct ways of self-care, being able to cause an increase in the quality of life of the cases with tracheostomy after the discharge from the hospital [**35**].

**Limitations and suggestions**

The limitations of the study included individual differences and different reasons of the subjects, which could have an effect on learning how to take care of them. On the other hand, the probability of using mass media, including radio and television or other educational resources were other limitations of this study which were out of control for the researcher but they could happen in both groups. It is suggested that future studies should assess and compare the efficacy of other educational methods regarding the quality of life of tracheostomy cases, the effect of audio–video materials on the incidence of complications and readmissions in this group of patients, and the use of education videos on the quality of life of other patients.

## Conclusion 

The results of this research indicated that the decrease in the quality of life of cases via tracheostomy occurred after discharge in the check team. Due to the increase in the quality of life of patients in the intervention group following the use of educational videos, the healthcare team, especially nurses, can use this training method additionally to routine care in order to improve the quality of life in cases with tracheostomy, as an educational program to use at home. 

**Acknowledgment**

This paper is the result of an MSc thesis in Critical Care Nursing in Tehran University of Medical Sciences. I would like to thank the research deputy of the University for the financial support, research associate of School of Nursing and Midwifery, patients and nurses in Amir Alam Hospital, Imam Khomeini Cancer Institution and also the research assistant of Kurdistan University of Medical Sciences.
